# Spectrum of malignant and premalignant skin lesions in 505 adult subjects at risk of skin cancers

**DOI:** 10.1186/s12885-024-12035-w

**Published:** 2024-03-14

**Authors:** Reetta Nevakivi, Hanna Siiskonen, Salla Haimakainen, Ilkka T. Harvima

**Affiliations:** Department of Dermatology, University of Eastern Finland and Kuopio University Hospital, Kuopio, Finland POB 100, 70029

**Keywords:** Malignant melanoma, Squamous cell carcinoma, Basal cell carcinoma, Actinic keratosis, Platelet-to-lymphocyte ratio, Photodamage

## Abstract

Patients at risk of skin cancers can develop varying types of cutaneous malignancies. However, some subjects may develop only one type of lesion. In this cross-sectional study, the spectrum of premalignant (PM) and malignant skin lesions and their risk factors were studied. Therefore, 505 adult subjects (aged 21–79 years, 256 males and 249 females, 96 with immunosuppression) at risk of any type of skin cancer were examined for cutaneous malignancies, nevi, actinic keratoses, photodamage, and possible risk factors. First, 12 different groups were identified with a varying set of PM and/or malignant skin lesions. Next, 5 larger groups were formed from them: basal cell carcinoma (BCC) only, malignant melanoma (MM) only, squamous cell carcinoma (SCC) and/or PM, BCC + SCC and/or PM, and MM + keratinocyte carcinoma (KC) and/or PM. The groups with BCC or MM only were younger and showed less photodamage than the mixed groups, while SCC/PM showed similarity with them. In logistic regression analyses, the platelet-to-lymphocyte ratio was associated with an increased risk of concomitant KC (OR 1.028, *p* = 0.023) or SCC/PM (OR 1.009, *p* = 0.047) in subjects with MM or BCC, respectively. Actinic keratoses produced ORs 0.246–0.252 (*p* = 0.008–0.020) for BCC in subjects with SCC/PM. Interestingly, atypical mole syndrome decreased the risk of SCC/PM in subjects with BCC (OR 0.092, *p* = 0.001). Advanced age was a significant risk factor for an additional type of lesion in all 3 comparisons (ORs 1.088–1.388, *p* = 0.001). In conclusion, even though there are numerous patients with only one lesion type, advancing age may determine the final lesion multiplicity.

## Introduction

During the last decades, the incidences of malignant melanoma (MM) and keratinocyte skin cancers (KC), i.e., basal cell carcinoma (BCC) and squamous cell carcinoma (SCC), have been increasing in all Western countries owing to increased exposure of skin to solar ultraviolet (UV) light [1]. Patients with skin cancer are known to be at increased risk of developing a subsequent skin cancer of the same or another type, largely due to shared risk factors, such as UV radiation. Other shared risk factors, such as immunosuppression and genetics, may also play a role in the risk of developing multiple cutaneous malignancies [2]. In addition, the prognosis of cutaneous malignancies is generally quite favorable, which makes the development of multiple skin cancers in the same patient possible.

Numerous previous studies have reported the coexistence of different types of keratinocyte skin carcinomas, melanoma and premalignant lesions in the same patient [3–5]. Rarely, melanoma and keratinocyte skin carcinomas can even be present in the same skin tumor [6]. Therefore, these skin lesions are closely interrelated to each other, even though differences exist in their genetic, phenotypic and environmental risk factors. Moreover, prior KC has been found to be a strong predictor of subsequent KC, and it has also been noted that KC patients tend to develop KCs of the same type as the previous KC. This was also seen in a meta-analysis published in 2013, in which BCC patients’ pooled standardized incidence ratios (SIRs) for subsequent BCC, SCC or MM were 17.4, 3.2 and 2.4, respectively. In SCC patients, the SIRs were 4.2, 15.0, and 2.7, respectively [5]. In an Australian population-based cohort study, 1621 subjects with no previous KC were followed up to 16 years for the emergence of KC. During the follow-up, 21% of the patients developed one KC, and 47% of those patients developed at least 2 KCs. Of the people who developed multiple KCs, 56% exclusively had BCCs (ranging from 2–8 BCCs per person), 16% exclusively SCCs (2–5 per person) and 28% had a combination of both types [7].

Most of the previous studies have been conducted with data from national cancer registries. However, the registries rarely contain information on premalignant (PM) or in situ lesions, that is, actinic keratosis (AK), SCC in situ, also called Bowen’s disease (BD), or melanoma in situ. Nonetheless, it has been previously shown that most patients with SCC have a history of previous PM lesions [8]. SCC can arise de novo or within a PM lesion, and several studies have reported that the majority of SCCs arise within preexisting AKs or in their immediate proximity [9]. Although AK and BD only progress into SCCs, the presence of these PM lesions is a strong predictor of skin carcinogenesis associated with the risk of developing not only SCC but also BCC and, to a lesser extent, MM [10]. The risk of an individual AK lesion progressing into SCC ranges from 0% to 0.075% per lesion-year, but in patients with previous KC, the rate of progression is higher, up to 0.53%. The risk of an individual BD developing into invasive SCC is higher and is estimated to range from 3 to 5% [11]. Although the progression from AK into SCC is possible with time when left untreated, e.g. in actinic field damage, many of the AK lesions do not show malignant progression. Instead, the rate of regression of an individual lesion ranges between 15 and 63% after one year [12].

Previously, it has been suggested that there can be subjects among skin cancer patients who develop only one type of skin cancer, such as BCC or SCC [7]. To obtain further understanding of the patterns of malignant and PM skin lesions and their risk factors, 505 adult subjects at risk of any type of skin cancer were interviewed and examined for past or present skin cancers or malignancies in the extracutaneous sites, AKs, pigment-cell nevi, photodamage severity, and several possible risk factors related to skin cancers.

## Materials and methods

### Study subjects

In this study, 505 adult subjects (aged 21–79 years, mean ± SD age 62.4 ± 13.7 years, 256 males and 249 females) were recruited at the dermatological outpatient clinic of Kuopio University Hospital, Kuopio, Finland, from May 2017—October 2020. For the recruitment, the medical staff in the outpatient clinic, unrelated to the study, were instructed to provide all subjects aged 18 to 80 years referred to the clinic with a possibility to participate in the study based on the preliminary assessment of their risk of developing any type of skin cancer, including the evaluation of the history of skin cancers or PM lesions, cutaneous photodamage, number of moles, atypical moles, immunosuppression, skin phototype, and family history of melanoma, as described recently [13–15]. Upon arrival, the subject declared his or her willingness to participate in the study. Each subject completed a questionnaire about demographic details, personal skin cancer history, family history of skin cancers, personal medical history related to diseases or malignancies in internal organs, and other factors connected to skin cancers, such as a history of UV exposure, Fitzpatrick skin type, tobacco smoking and alcohol use.

All study subjects signed an informed consent before entering the study. The study was approved by the Ethics Committee of Kuopio University Hospital (number of approval 71/2017), adhering to the principles outlined in the declaration of Helsinki. All data obtained from the study participants were handled in compliance with the General Data Protection Regulation of the European Union.

The evaluation of the immunosuppression status was based on marked immunosuppressive medication in OTRs or in subjects with immune-mediated diseases during the past years, as described by Komulainen et al. [13], and 96 subjects (53 males and 43 females) were judged to be immunosuppressed (39 OTRs). In addition, there was one OTR subject who had received bone marrow transplant and was therefore not immunosuppressed.

The subjects underwent dermatologic examination of the entire skin. The numbers of AKs and pigment-cell nevi were counted. Consequently, the subjects with AKs were divided into 6 groups: 1) 0; 2) 1; 3) 2; 4) 3; 5) 4–10; and 6) > 10 AKs. The subjects with nevi were divided into 4 groups: 1) 0–20; 2) 21–50; 3) 51–100; and 4) > 100 nevi. Diagnostic biopsies were taken if necessary [13]. The subjects were also examined for the presence of atypical mole syndrome (AMS). The diagnostic criteria of AMS vary, but typical features include the abundance of melanocytic nevi (over 50), their heterogeneous appearance and localization to usual and unusual body sites, and the presence of clinically atypical nevi [16, 17].

The PAASI (PhotoAging Area and Severity Index) score was developed recently for the assessment of the photodamage severity of the entire skin. The skin sites examined were the face and ears (area percentage 3%), upper scalp (2%), the rest of scalp (2%), neck and collum (3%), dorsal aspects of hands and wrists (2%), dorsal aspects of forearms (4%), dorsal aspects of arms and shoulders (5%), medial aspects of upper limbs (9%), upper back (5%), middle and lower back (9%), upper chest (5%), middle chest and abdomen (11%), legs and dorsal feet (13%), thighs (19%), buttocks (5%), and soles (3%). The skin sites were evaluated using the following scoring: 0 = no apparent photodamage (intrinsic skin aging), 1 = mild photodamage, 2 = moderate photodamage, 3 = severe photodamage with AK, and 4 = very severe photodamage with several AKs. The scores of individual skin sites were multiplied by their area percentage and added together, forming a score ranging from 0 to 400.

Fitzpatrick skin type was self-reported on a scale from I-VI. Additionally, subjects filled an 8-point Fitzpatrick questionnaire (adopted from The Skin Cancer Foundation, New York) on their pigmentary characteristics (eye, hair and skin color), freckling, ability to tan, propensity to burn and facial sensitiveness towards UV exposure. Each question was scored 0–4, and the total score ranged from 0 to 30.

### Blood tests

A venous blood sample was taken from the subjects and analyzed in the hospital laboratory (ISLAB) of Kuopio University Hospital. The level of hemoglobin, thrombocytes, and white blood cell count and differential were analyzed. Previous studies have shown that the numerical ratios of different blood cells, such as the neutrophil-to-lymphocyte ratio (NLR), eosinophil-to-lymphocyte ratio (ELR) and platelet-to lymphocyte ratio (PLR), can be used as simple and inexpensive markers of systemic inflammation, and elevated values have also been linked to cancer incidence and prognosis. Therefore, these values were also calculated [18–20].

### Statistics

Statistical analyses were conducted using IBM SPSS Statistics for Windows, Version 27.0. (Armonk, NY: IBM Corp). The differences between continuous variables were tested using the Mann‒Whitney test or Kruskal‒Wallis test, and the differences between categorical variables were tested using the chi-square test or Fisher’s exact test. The risk factors for different skin lesion combinations were analyzed using binomial logistic regression analysis. Crude ORs were calculated for each factor. Multivariate models were formed by combining variables that produced statistically significant univariate ORs and then adding nonsignificant but clinically relevant or otherwise interesting variables. ROC analyses were used to test the fit of the models. A *p*-value less than 0.05 was considered statistically significant.

## Results

### Spectrum of premalignant and malignant skin lesions

Of the total 505 subjects, 297 (58.8%) had past or present skin cancer and 282 (55.8%) past or present PM lesions, meaning AKs and BDs, and 100 subjects (19.8%) had no history of these skin lesions (Table 1).

**Table 1:** The characteristics of 405 subjects with PM or malignant skin lesions divided into 12 groups according to skin lesion combinations

	**No skin cancer, no PM *n* = 100**	**PM only *n* = 108 (AK 107, BD + AK 1)**	**BCC only *n* = 65**	**SCC only *n* = 1**	**MM only *n* = 48 (40 MM, 8 LM/MIS)**	**SCC + PM *n* = 10 (AK 9, BD + AK 1)**	**BCC + PM *n* = 102 (AK 99, BD + AK 3)**	**MM + PM *n* = 21 (MM 16, LM/MIS 5, AK 20, BD + AK 1)**	**MM + BCC *n* = 9 (MM 9, LM/MIS 0)**	**MM + SCC + PM *n* = 4 (MM 4, LM/MIS 0)**	**MM + BCC + PM *n* = 15 (MM 14, LM/MIS 1)**	**BCC + SCC + PM *n* = 17 (AK 15, BD + AK 2)**	**MM + BCC + SCC + PM *n* = 5 (MM 3, LM/MIS 2, AK 4, BD + AK 1)**	***P*-value**
Proportion in 505 subjects	19.8%	21.4%	12.9%	0.2%	9.5%	2.0%	20.2%	4.2%	1.8%	0.8%	3.0%	3.4%	1.0%	-
Proportion in 405 subjects with PM and/or skin cancers	excluded	26.7%	16.0%	0.2%	11.9%	2.5%	25.2%	5.2%	2.2%	1.0%	3.7%	4.2%	1.2%	
Age, years (*n* = 505)														
range	21-75	43-79	31-79		27-73	58-79	40-79	57-79	44-79	69-77	52-79	63-78	67-76	**< 0.001**
mean ± SD	47.1 ± 14.0	67.6 ± 7.8	61.4±12.9	67.5	56.1±11.1	72.4±5.8	69.9±7.0	69.1±7.2	66.6±11.3	72.6±3.4	70.2±7.5	71.7±4.8	72.1±4.5	
Sex(*n*=505)														
Male (%)	45 (45.0)	59 (54.6)	23 (35.4)	0 (0.0)	23 (47.9)	6 (60.0)	59 (57.8)	10 (47.6)	5 (55.6)	3 (75.0)	8 (53.3)	11 (64.7)	4 (80.0)	0.204
Female	55	49	42	1	25	4	43	11	4	1	7	6	1	
Immunosuppression *n* = 505)														
No	63	90	56	1	45	8	87	16	9	4	14	12	4	0.406
Yes (%)	37 (37.0)	18 (16.7)	9 (13.8)	0 (0.0)	3 (6.3)	2 (20.0)	15 (14.7)	5 (23.8)	0 (0.0)	0 (0,.0)	1 (6.7)	5 (29.4)	1 (20.0)	
Organ transplant recipients, n (%)	22 (22.0)	5 (4.6)	3 (4.6)	0 (0.0)	0 (0.0)	0 (0.0)	5 (4.9)	1 (4.8)	0 (0.0)	0 (0.0)	0 (0.0)	2 (11.8)	1 (20.0)	0.456
PAASI score (mean±SD)(*n* = 503)	30.5±31.4	78.4±36.4	53.0±31.7	90.0	56.6±38.4	95.3±55.6	85.0±39.6	78.6±36.9	82.4±48.8	104.3±87.9	92.9±31.9	104.1±67.3	91.8±42.7	**<0.001**
Fitzpatrick skin type (*n* = 475)														
I	3	5	1	0	3	0	6	1	1	0	4	0	0	0.103
II	41	36	27	1	21	5	40	10	3	1	4	9	2	
III	50	57	30	0	19	2	48	9	4	1	7	3	1	
IV	2	5	2	0	2	0	3	1	0	2	0	2	1	
Number of AKs (*n* = 504) (%)														
0	100 (100)	18 (16.8)	65 (100.0)	1 (100)	48 (100)	1 (10.0)	26 (25.5)	6 (28.6)	9 (100)	0 (0.0)	3 (20.0)	3 (17.6)	2 (40.0)	** < 0.001**
1–3	0 (0.0)	46 (43.0)	0 (0.0)	0 (0.0)	0 (0.0)	5 (50.0)	42 (41.2)	10 (47.6)	0 (0.0)	1 (25.0)	8 (53.3)	5 (29.4)	0 (0.0)	
4–10	0 (0.0)	27 (25.2)	0 (0.0)	0 (0.0)	0 (0.0)	2 (20.0)	21 (20.6)	3 (14.3)	0 (0.0)	2 (50.0)	1 (6.7)	5 (29.4)	2 (40.0)	
over 10	0 (0.0)	16 (15.0)	0 (0.0)	0 (0.0)	0 (0.0)	2 (20.0)	13 (12.7)	2 (9.5)	0 (0.0)	1 (25.0)	3 (20.0)	4 (23.5)	1 (20.0)	
Number of moles (n = 503) (%)														
0–20	29 (29.0)	73 (67.6)	33 (50.8)	1 (100)	8 (16.7)	9 (90.0)	58 (57.4)	11 (52.4)	2 (22.2)	2 (50.0)	8 (52.2)	10 (62.5)	0 (0.0)	** < 0.001**
21–50	21 (21.0)	20 (18.5)	13 (20.0)	0 (0.0)	18 (37.5)	0 (0.0)	30 (29.7)	3 (14.3)	3 (33.3)	1 (25.0)	5 (33.3)	2 (12.5)	2 (40.0)	
21–50	25 (25.0)	8 (7.4)	13 (20.0)	0 (0.0)	12 (25.0)	1 (10.0)	8 (7.9)	5 (23.8)	2 (22.2)	0 (0.0)	1 (6.7)	3 (18.8)	2 (40.0)	
51–100	25 (25.0)	7 (6.5)	6 (9.2)	0 (0.0)	10 (20.8)	0 (0.0)	5 (5.0)	2 (9.5)	2 (22.2)	1 (25.0)	1 (6.7)	1 (6.3)	1 (20.0)	

Patients with no skin cancer or PM lesions excluded from the analyses. The differences between continuous variables were tested using Kruskal–Wallis test. The categorical variables were tested with chi-square or Fisher’s exact test. The statistically significant values are in bold

*Abbreviations*: *PM* Premalignant, *AK* Actinic keratosis, *BD* Bowen’s disease, *BCC* Basal cell carcinoma, *SCC* Squamous cell carcinoma, *MM* Malignant melanoma, *LM* Lentigo maligna, *MiS* Melanoma in situ, *PAASI* PhotoAging Area and Severity Index

Of the 297 subjects with a past or present skin cancer, there were 102 subjects with MM, 213 with BCC, 37 with SCC, 247 (83.2%) with only one type of skin cancer (167 BCC, 69 MM and 11 SCC), and 50 (16.8%) with skin cancers of more than one type. The most common tumor combination was MM and BCC (24 subjects), followed by 17 subjects with BCC and SCC and 4 with MM and SCC. Five of the subjects showed a combination of all three types of skin cancer, i.e., MM, BCC and SCC.

**Fig. 1:**
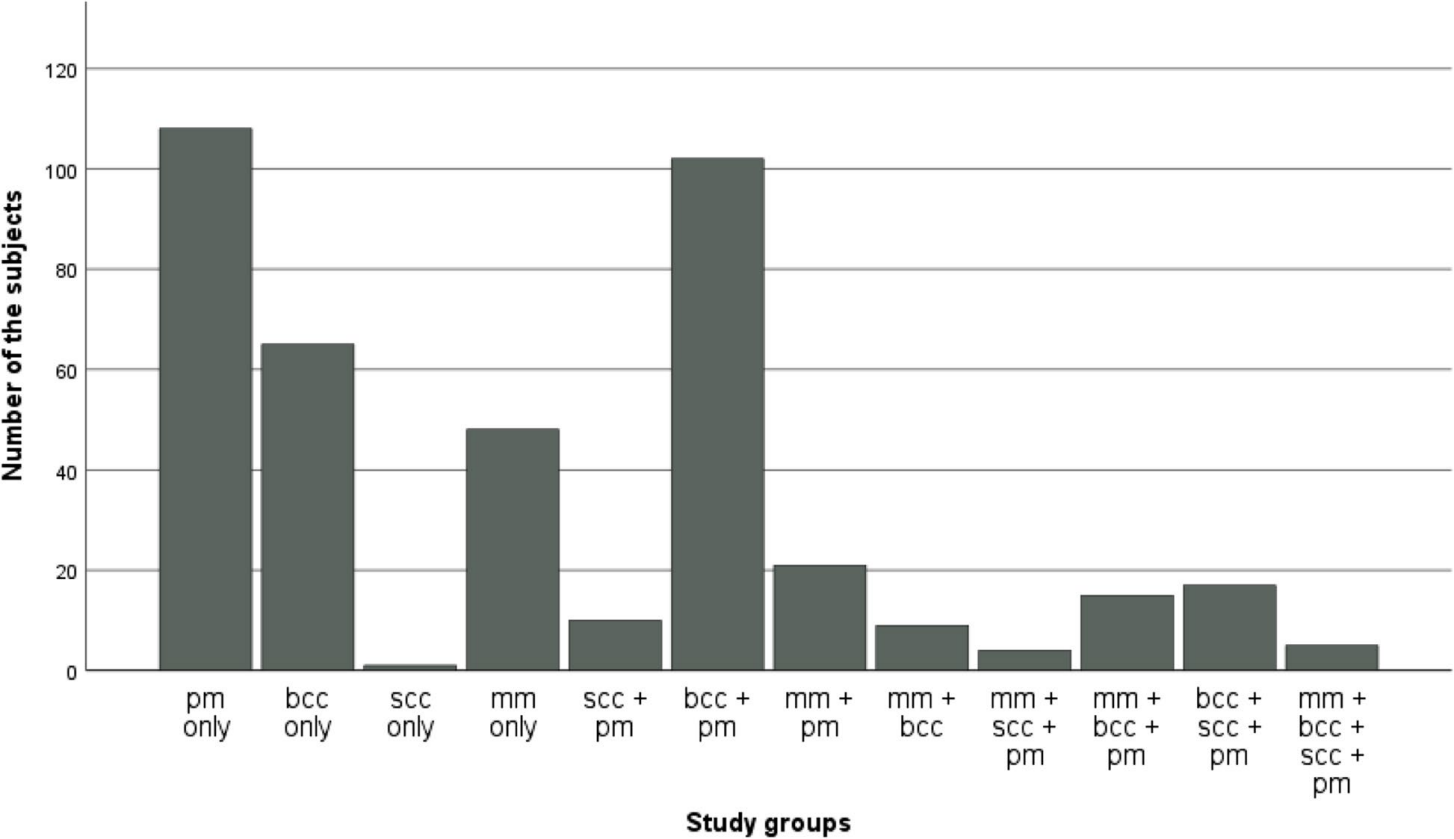
Distribution of 405 subjects across 12 groups based on premalignant and malignant skin lesions. *Abbreviations*: PM premalignant, BCC basal cell carcinoma, SCC squamous cell carcionoma, MM malignant melanoma

By taking into consideration PM lesions, the study subjects with a PM and/or malignant lesions were divided into 12 different groups, that is, one subject was present in one group only (Table 1, Fig. 1). Among them, 108 subjects constituted the largest group with PM lesions only (26.7%), followed by a group with BCC and PM lesions (102 subjects, 25.2%), BCC only (65 subjects, 16.0%) and MM only (40 with MM and 8 with lentigo maligna (LM)/melanoma in situ (MIS), 11.9%). Only 17 subjects (4.2%) revealed a history of a combined KC line (BCC, SCC and PM), but none had a history of both BCC and SCC without any other lesions. Of note, there were only 11 subjects (0.3%) with exclusively SCC line lesions (1 with SCC and 10 with SCC and PM). In combined lesions with MM, MM was most frequently seen together with PM (21 subjects, 5.2%), followed by 15 subjects with MM, BCC and PM (3.7%), 9 with MM and BCC (2.2%), 5 with MM, BCC, SCC and PM (1.2%), and 4 with MM, SCC and PM (1.0%). Due to the low number of cases in these combined groups with MM, no definite conclusions can be drawn from the relative distribution of subjects with either MM or LM/MIS. However, it seems that there are no clear differences.

### Comparison between the 12 groups

As expected, the 100 control subjects without any PM or malignant skin lesions were young (47.1 ± 14.0 versus 66.3 ± 10.1 years) and consequently showed low PAASI but a high frequency of subjects with immunosuppression (37.0% vs. 14.6%) or organ transplant (22% vs. 4.2%).

With regard to the age in the other 12 groups with skin lesions, there were 2 groups with relatively low mean age, i.e., subjects with BCC only (61.4 ± 12.9) and those with MM only (56.1 ± 11.1) (*p* < 0.001 and *p* < 0.001, respectively, when compared to the mean age of the other 10 groups). In addition, the group with BCC only revealed a clearly lower male/female ratio than the other 11 groups (23/42 vs. 188/152, *p* = 0.003). No significant differences were detected in the immunosuppression status (Table 1) or BMI (not shown) between the groups.

Together with the relatively low mean age, the PAASI score was relatively low in the group with BCC or MM only. The highest PAASI scores were typically seen in the groups with SCC line lesions, all of whom had a mean score of at least 90. However, due to small group sizes, significance was only reached in the groups with BCC and PM lesions (85.0 ± 39.6 versus 72.9 ± 42.4, *p* = 0.003) and BCC, MM and PM lesions (92.9 ± 31.9 vs. 75.3 ± 42.3, *p* = 0.037).

Pigmentary traits were assessed using Fitzpatrick skin type (Table 1) and score (not shown). None of the groups had a significantly different Fitzpatrick score compared to the rest, except for the control group, which showed a significantly higher mean score than the rest of the groups (15.5 ± 4.8 vs. 14.1 ± 4.5, *p* = 0.012). Some slight differences in the proportions of different skin types were seen in the groups with a low number of cases, but in the larger groups, the distribution was similar, including the control group.

As expected, large differences in the distribution of the number of present AKs were observed, as some of the groups contained only subjects without PM lesions (Table 1). However, there were no differences in this distribution when comparing the group with PM lesions only to those with BCC and PM or MM and PM. With respect to the distribution of nevus count, the striking difference was the high frequency of subjects with high nevus count in the group with MM only (Table 1).

### Comparison between the 5 combined groups

Next, the subjects (excluding the control group) were divided into 5 larger groups for more detailed comparisons (Table 2). Subjects with SCC alone or together with PM were combined with those with PM alone, producing the SCC group with 119 subjects. The 65 subjects with BCC only constituted the BCC group. The mixed group of both BCC and SCC line (SCC and/or PM) lesions consisted of 119 subjects. The 48 subjects with MM only were also compared to the 54 subjects with MM and KC and/or PM (BCC, SCC and/or PM).

**Table 2:**
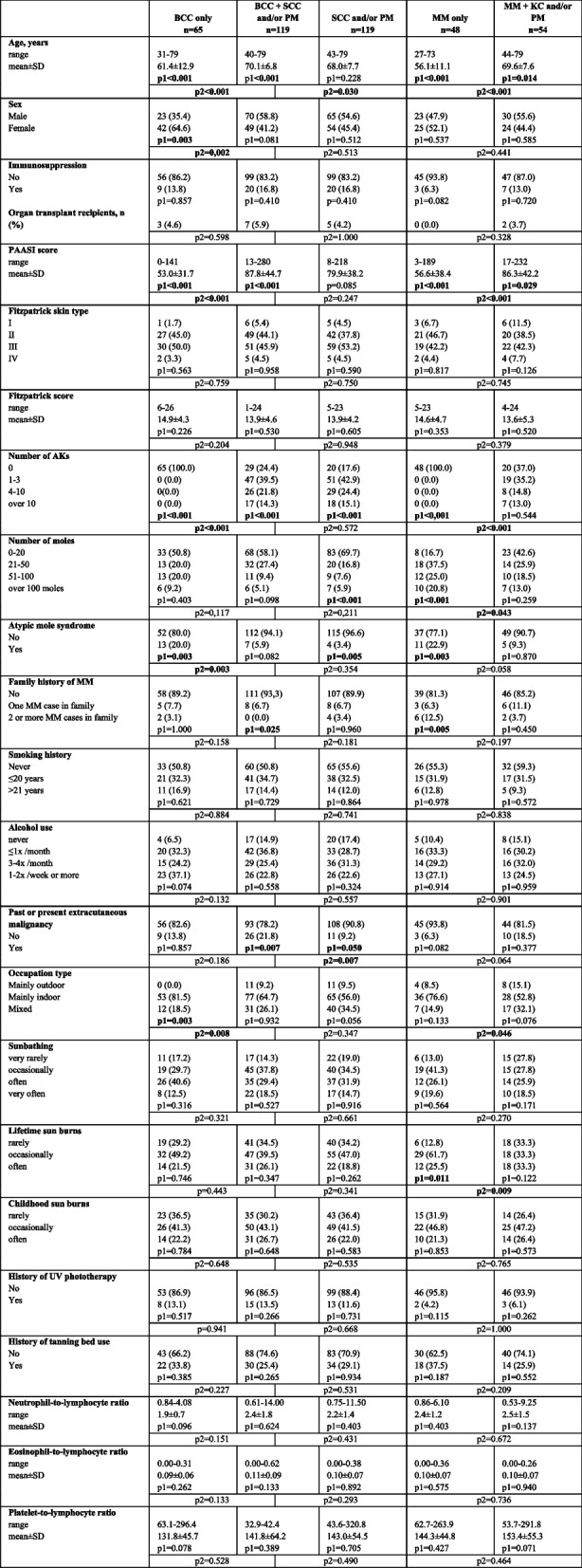
Comparisons between the individual groups and the rest of the subjects, and pairwise comparisons of the groups

The p1-values shown refer to the comparison between individual groups and the rest of the subjects, and the p2 values refer to the pairwise comparisons between the groups. The differences between continuous variables were tested using Mann–Whitney test. The categorical variables were tested with chi-square or Fisher’s exact test. Significant values bolded

*Abbreviations*: PM premalignant; BCC basal cell carcinoma; SCC squamous cell carcinoma; MM malignant melanoma; PAASI PhotoAging Area and Severity Index; AK actinic keratosis; UV ultraviolet

As also presented above, the mean age was relatively low in the group with BCC or MM only compared to other groups, and the male/female ratio was strikingly low among subjects with BCC only. No significant differences were observed in immunosuppression status, Fitzpatrick skin type, smoking, alcohol usage, history of UV exposure, UV phototherapy or tanning bed use. However, together with the higher age, the subjects with both BCC and SCC/PM showed a more frequent outdoor working history and a higher PAASI score than the subjects with BCC only. Of note, there was no difference in nevus count, but the proportion of subjects with AMS was significantly higher in the group with BCC only (20.0% vs. 5.9%, *p* = 0.003) than in the group with BCC and SCC/PM.

Subjects in the group with MM only reported more often lifetime sunburns compared to other groups (87.2% vs. 66.7% reported having had sunburns occasionally or often, *p* = 0.011), yet no differences were seen in the sunburn history of childhood. Furthermore, there were more frequent subjects with high nevus counts but with lower PAASI scores in this group with MM only than in MM subjects with concomitant KC/PM, although there was no difference in the family history of melanoma. The number of AMS cases was higher in the group with MM only (22.9%) than in the MM and KC/PM groups (9.3%), but only borderline significance was reached (*p* = 0.058).

The group with SCC line lesions was similar to the group with both BCC and SCC/PM or that with both MM and KC/PM with respect to age, sex, immunosuppression status, Fitzpatrick skin type, PAASI, family history of melanoma, smoking, alcohol usage, working environment history, and lifetime UV exposure. Subjects with MM and KC/PM more frequently showed a high nevus count but fewer AKs counted at study entry than subjects with SCC line lesions only.

Malignancies in extracutaneous sites were signifincantly more common in the group with both BCC and SCC/PM, where 21.8% (*p* = 0.007) of the subjects had a past or present history of extracutaneous malignancy, compared to other groups with clearly lower percentages.

No significant differences were observed in the blood biomarkers of inflammation or cancer (NLR, ELR or PLR). When comparing subjects with one type of cancer to subjects with more than one type of skin cancer (not shown in tables), the NLR was significantly higher in subjects with at least two different skin cancers (2.2 ± 1.1 vs. 2.9 ± 2.4, χ^2^ *p* = 0.041) and showed a significant age- and sex-adjusted OR in logistic regression analysis, OR 1.29 (*p* = 0.013, 95% CI 1.056–1.577).

### Binary logistic regression analysis

Binary logistic regression analysis was performed to identify the factors that may independently affect the risk for SCC/PM or KC/PM lesions in subjects with BCC or MM, respectively, as well as the factors possibly affecting the risk of BCC in subjects with SCC and/or PM lesions. For each of these three comparisons, univariate ORs were first calculated for each variable (only variables selected for the final models are shown in Tables 3, 4 and 5). Multivariate regression models were formed combining variables with significant univariate ORs and variables presumed to have clinical relevance or otherwise of specific interest, such as PLR, which showed clear yet nonsignificant differences between groups in the preceding analyses. The results of univariate and multivariate models are shown in Tables 3, 4 and 5.

**Table 3:** The logistic regression analysis and consequent odds ratios for subjects with BCC and SCC and/or PM lesions compared to subjects with BCC only

	**Univariate OR**	***p*-value**	**95% CI**	**Multivariate OR**	***p*-value**	**95% CI**
Age years	1.098	** < 0.001**	1.057–1.142	1.088	** < 0.001**	1.038–1.140
Sex						
Male	1 (ref)			1 (ref)		
Female	0.383	**0.003**	0.205–0.717	0.210	**0.002**	**0.002**
Immunosuppression						
No	1 (ref)			1 (ref)		
Yes	1.257	0.599	0.536–2.948	1.077	0.894	0.359–3.229
Past or present extracutaneous malignancy						
No	1 (ref)			1 (ref)		
Yes	1.740	0.190	0.761–3.979	1.374	0.572	0.457–4.133
Occupation type						
mainly indoor	1 (ref)			1 (ref)		
mainly outdoor/mix	2.409	**0.018**	1.160–5.003	1.183	0.756	0.409–3.424
Lifetime sunburns						
Rarely	1 (ref)			1 (ref)		
Occasionally	0.681	0.285	0.336–1.378	1.841	0.215	0.701–4.833
Often	1.026	0.952	0.446–2.361	2.872	0.058	0.965–8.546
ASA use						
no use	1 (ref)			1 (ref)		
occasional	1.243	0.738	0.347–4.459	1.612	0.587	0.288–9.032
regular	3.350	**0.008**	1.380–8.133	2.405	0.121	0.794–7.285
Alcohol consumption						
never	1 (ref)			1 (ref)		
≤ 1x /month	0.494	0.254	0.147–1.661	1.049	0.948	0.248–4.443
3-4x /month	0.455	0.219	0.130–1.596	0.545	0.417	0.125–2.364
1-2x/week or more	0.266	**0.034**	0.078–0.906	0.189	**0.028**	0.043–0.837
Platelet-to-lymphocyte-ratio	1.003	0.276	0.998–1.009	1.009	**0.047**	1.000–1.018
AMS						
no	1 (ref)			1 (ref)		
yes	0.250	**0.005**	0.094–0.663	0.092	**0.001**	0.022–0.396

The multivariate ORs adjusted for all covariates presented in the table. The statistically significant values are in bold. *Abbreviations:*CI, confidence interval; PM premalignant; BCC basal cell carcinoma; SCC squamous cell carcinoma; AMS atypic mole syndrome; ASA acetylsalicylic acid

In the model comparing subjects with BCC and SCC/PM to control subjects with BCC only (Table 3), age produced a significant OR in both univariate and multivariate analyses, with a multivariate OR of 1.088. Similarly, female sex and alcohol consumption > 1–2 times a week compared to no use showed significant ORs in both univariate and multivariate models, with multivariate ORs of 0.210 and 0.189, respectively. Outdoor/mixed working environment compared to indoor work and regular acetylsalicylic acid (ASA) use compared to no use showed significantly increased ORs in the univariate analyses but remained nonsignificant in the multivariate analysis. In the multivariate model, the ascending PLR ratio increased the risk of SCC/PM in BCC subjects (OR 1.009), whereas the presence of AMS decreased it (OR 0.092). Other variables showed no significant ORs.

When comparing subjects with MM and KC/PM to control subjects with MM only (Table 4), age again produced significant ORs in both univariate and multivariate models, with a multivariate OR of 1.388. An opposite significance was seen in the history of sunburn; the multivariate OR for sunburn “occasionally” compared to “rarely” was 0.038 (*p* = 0.007). Significant associations in univariate analyses were detected in the following variables: outdoor/mixed working environment compared to indoor work (OR 2.922), nevus count (OR 0.243–0.290), regular use of ASA (OR 3.152), and occasional use of NSAIDs compared to no use (OR 0.382). However, except for the occasional use of ASA (OR 0.010), all of them produced nonsignificant ORs in the multivariate analysis. Interestingly, the PLR ratio showed a significant association with KC/PM in the multivariate analysis with an OR of 1.028.

**Table 4:** The logistic regression analysis and consequent odds ratios for subjects with MM and KC and/or PM lesions compared to subjects with MM only

	Univariate OR	*P*-value	95% CI	Multivariate OR	*P*-value	95% CI
Age years	1.169	** < 0.001**	1.099–1.242	1.388	** < 0.001**	1.163–1.656
Sex						
Male	1 (ref)	0.441	0.337–1.606	1 (ref)		
Female	0.736			5.653	0.144	0.554–57.715
Immunosuppression						
No	1 (ref)	0.265	0.544–9.177	1 (ref)	0.303	0.161–354.972
Yes	2.234			7.570		
Occupation type						
mainly indoor	1 (ref)	0.015	1.231–6.934	1 (ref)	0.350	0.353–18.937
mainly outdoor/mix	2.922			2.586		
Lifetime sunburns						
rarely	1 (ref)			1 (ref)		
occasionally	0.207	**0.005**	0.069–0.619	0.038	**0.007**	0.004–0.401
often	0.500	0.249	0.154–1.624	4.068	0.311	0.269–61.560
Number of moles						
0–20	1 (ref)			1 (ref)		
21–50	0.271	**0.016**	0.093–0.785	0.111	0.071	0.010–1.204
51–100	0.290	**0.037**	0.091–0.927	1.563	0.723	0.132–18.526
over 100 mol	0.243	**0.028**	0.069–0.856	2.967	0.617	0.042–210.301
ASA use						
no	1 (ref)			1 (ref)		
occasional	0.697	0.639	0.154–3.153	0.010	**0.031**	0.000–0.666
regular	3.152	**0.023**	1.170–8.489	4.610	0.214	0.414–51.354
NSAID use						
no	1 (ref)			1 (ref)		
occasional	0.382	**0.028**	0.162–0.900	0.572	0.591	0.075–4.372
regular	0.441	0.269	0.104–1.881	0.062	0.135	0.002–2.374
Platelet-to-lymphocyte-ratio	1.004	0.371	0.996–1.012	1.028	**0.023**	1.004–1.053
Family history of MM						
No	1 (ref)			1 (ref)		
One MM case in family	1.696	0.475	0.398–7.229	50.273	0.078	0.645–3916.795
2 or more MM cases	0.283	0.135	0.054–1.481	0.062	0.098	0.002–1.670
AMS						
no	1 (ref)			1 (ref)		
yes	0.343	0.066	0.110–1.073	0.060	0.119	0.002-2.056

The multivariate ORs adjusted for all covariates presented in the table. The statistically significant values are in bold. *Abbreviations:*CI, confidence interval; MM, melanoma; KC, keratinocyte carcinoma; PM premalignant; NSAID, non-steroidal anti-inflammatory agent; AMS atypic mole syndrome; ASA acetylsalicylic acid

In the comparison of subjects with BCC and SCC/PM to control subjects with SCC/PM only (Table 5), advanced age, past or present malignancy in extracutaneous sites and nevus count of 21–50 compared to 0–20 showed significant associations in both univariate and multivariate comparisons, with multivariate ORs of 1.099, 2.839 and 2.557, respectively. The variables of “main working environment” and “number of AKs” showed no significance in the univariate analyses, but significant associations were seen in the multivariate regression, where the outdoor/mixed environment decreased the risk of BCC (OR 0.507) compared to indoor occupation, as did 4–10 AKs (OR 0.246) and over 10 AKs (OR 0.252) compared to no AKs. Of note, the PLR ratio did not reveal any significance in this analysis.

**Table 5:** The logistic regression analysis and consequent odds ratios for subjects with BCC and SCC and/or PM lesions compared to subjects with SCC and/or PM

	**Univariate OR**	***P*-value**	**95% CI**	**Multivariate OR**	***P*-value**	**95% CI**
Age years	1.042	**0.026**	1.005–1.081	1.099	**0.001**	1.040–1.160
Sex						
Male	1 (ref)			1 (ref)		
Female	0.843	0.513	0.504–1.408	0.560	0.110	0.275–1.140
Immunosuppression						
No	1 (ref)			1 (ref)		
Yes	1.000	1.000	0.507–1.973	1.118	0.807	0.456–2.738
Past or present extracutaneous malignancy					**0.028**	1.117–7.219
No	1 (ref)			1 (ref)		
Yes	2.745	**0.009**	1.287–5.855	2.839	**0.028**	1.117–7.219
Occupation type						
mainly indoor	1 (ref)			1 (ref)		
mainly outdoor/mix	0.695	0.175	0.411–1.175	0.507	**0.049**	0.258–0.998
Lifetime sunburns						
rarely	1 (ref)			1 (ref)		
occasionally	0.834	0.542	0.465-1.495	0.921	0.817	0.457–1.856
often	1.375	0.372	0.684–2.765	2.045	0.092	0.889–4.700
Number of moles						
0–20	1 (ref)			1 (ref)		
21–50	1.9531	**0.042**	1.026–3.719	2.557	**0.018**	1.175–5.565
51–100	1.492	0.403	0.584–3.809	1.132	0.821	0.388–3.307
over 100 moles	1.046	0.938	0.336–3.260	1.624	0.505	0.390–6.775
Alcohol consumption						
never	1 (ref)			1 (ref)		
≤ 1x /month	1.497	0.317	0.679–3.303	2.375	0.076	0.914–6.174
3-4x /month	0.948	0.897	0.421–2.132	1.125	0.822	0.404–3.131
1-2x/week or more	1.176	0.706	0.506–2.738	1.019	0.974	0.343–3.023
Platelet-to-lymphocyte-ratio	1.000	0.880	0.995–1.004	0.999	0.853	0.994–1.005
Number of AKs diagnosed at the visit						
0	1 (ref)			1 (ref)		
1–3	0.636	0.201	0.318–1.272	0.524	0.138	0.223–1.231
4–10	0.618	0.226	0.284–1.346	0.246	**0.008**	0.088–0.691
over 10	0.651	0.336	0.272–1.561	0.252	**0.020**	0.079–0.807
PAASI score	1.005	0.152	0.998–1.011	1.003	0.399	0.996–1.011

The multivariate ORs adjusted for all covariates presented in the table. The statistically significant values are in bold

*Abbreviations:* *CI* Confidence interval, *PM* Premalignant, *BCC* Basal cell carcinoma, *SCC* Squamous cell carcinoma, *AK* Actinic keratosis, *PAASI* PhotoAging Area and Severity Index

## Discussion

This cross-sectional study demonstrates the heterogeneous spectrum of PM and malignant skin lesions, which is in line with previous studies showing that one type of skin cancer increases the risk of another type of skin cancer, including PM and in situ types of lesions [3–5]. The weaknesses of the study are that some of the information obtained from the patients, such as past UV exposure, may be subject to recall error, one patient may have developed multiple skin tumors of the same type over the past decades, and the study cohort does not represent the general population. The strength is that each patient underwent a thorough examination by an experienced dermatologist, including interviewing and investigation of medical records. Of note, the age of the study subjects refers to the age at entry to the study, not to that at skin cancer diagnosis.

With regard to the age and PAASI of subjects (Tables 1 and 2), an emerging finding was that the mean age (61.4) and PAASI (53.0) of subjects with BCC only were significantly lower when compared to corresponding values of subjects with BCC and PM (69.9 and 85.0, respectively) or those with BCC and SCC/PM (71.7 and 104), whereas the age (67.5–72.4) and PAASI (78.4–95.3) of the subjects with PM and/or SCC lesions were similar. This result suggests that the subjects with BCC only had not had the time to develop photodamage, PM and SCC, which is supported by the finding that subjects with BCC and SCC and/or PM revealed an outdoor working history significantly more often than subjects with BCC only. Alternatively, the difference in gender may explain this observation because the subjects with BCC only revealed a predominance to the female gender, but inversely, a predominance to the male gender was found in subjects with PM and/or SCC, with or without BCC. However, only the follow-up of these patients can reveal whether there are isolated groups that develop only BCC or SCC line lesions over time, as proposed by a previous Australian study [7]. As in the case of BCC only, the patients with MM only were relatively young (56.1), showing low PAASI (56.6), when compared to those in patients with MM and PM, BCC and/or SCC (age 66.6–72.6 and PAASI 78.6–104.3), whereas there was no apparent disparity in gender. This finding suggests, again, that the patients with MM only had not had the time for the carcinogenetic progression toward KCs, which is paralleled by the finding that there was a significantly more frequent outdoor working history in subjects with MM and KC and/or PM than in subjects with MM only. Another noticeable observation is that there were significantly more frequent nevi in younger subjects with MM only than in older subjects with MM and KC and/or PM, although some contradiction was noted in lifetime sunburns. The total number of nevi does not necessarily reflect the nevus- susceptibility of subjects because past UV exposure may have promoted nevus formation in a subject. Moreover, only 29.1% of melanomas originate from a preexisting nevus, and there are differences with regard to tumor characteristics between subjects with nevus-associated melanoma and those with de novo melanoma [21, 22]. However, this difference observed in nevi in the present study can imply that there are subjects with MM only that constitute an own subgroup with abundant moles that are prone to melanoma development, such as AMS, even though the difference in the frequency of AMS cases between these groups was of only borderline significance (*p* = 0.058). Previously, has been hypothesized that two distinct biological pathways of MM development exist: the nevus-prone pathway of young, nonactinically damaged individuals and the chronic sun exposure pathway of individuals less prone to nevi but with a more substantial history of UV exposure [23]. Nonetheless, only sufficiently long follow-up can confirm these conclusions.

In further logistic regression analyses, the independent risk factors for having SCC/PM in BCC subjects were advanced age, male sex, no use of alcohol and elevated PLR. Interestingly, the presence of AMS was associated with a lower risk of SCC/PM in BCC subjects. There appears to be no previous studies examining the connection between AMS and NMSC. However, the presence of melanocytic nevi, compared to no nevi, has been previously connected to increased BCC risk but not to SCC risk [24, 25]. Conversely, a recent study found an inverse association of numerous moles (number of moles 10 or more) and the risk of both SCC and BCC [26]. The magnitude of this effect was stronger in the SCC subjects (ORs 0.67–0.83) than in the BCC subjects (ORs 0.88–0.90) and is therefore in line with the present study connecting abundance of moles (AMS) to decreased SCC/PM risk [26]. Nevertheless, further follow-up of this connection is needed. Previous reports have shown that even though male sex is suggested to be an independent risk factor for BCC (as well as SCC), in younger age groups, BCC is more common in females [27]. Because the alcohol consumption has been found to be a risk factor for both BCC and cutaneous SCC, the decreased risk of SCC/PM in subjects with BCC also requires further follow-up [28].

Age and elevated PLR were independent risk factors for KC and/or PM in MM subjects. PLR, similar to NLR and ELR, is a simple blood biomarker that has previously been linked to systemic inflammation and cancer of many types [29]. Many studies have shown connections in these biomarkers in MM and cutaneous SCC, yet evidence on BCC is sparse [19, 30, 31]. Inflammation and immunity have commonly been accepted to play a role in carcinogenesis, and these blood biomarkers could serve as a minimally invasive and inexpensive method in skin cancer risk assessment. Unexpectedly, having experienced sunburns occasionally, rather than rarely, was independently associated with lower KC/PM risk in these MM subjects. Consistently, MM and BCC share a similar tendency to occur in patients with a history of intense, intermittent UV exposure, especially sunburns in childhood, but SCC has been viewed as a result of long-term, cumulative sun exposure [32]. Although no significant differences in the pigmentary traits between the groups were observed, it is possible that the subjects with MM only were more sensitive to sun and therefore experienced more sunburns, but the subjects with KC may be more sun tolerant, leading to cumulative actinic damage. Occasional ASA use was associated with a lower risk of KC and/or PM in melanoma subjects, which is in line with previous work associating ASA and/or nonsteroidal anti-inflammatory agents with reduced NMSC risk [33, 34]. However, this effect appears not to be dose-dependent because significance was not seen in the regular users of ASA.

In subjects with SCC/PM, advanced age, past or present extracutaneous malignancies and nevi were connected to elevated BCC risk. Previously, an association between NMSC and malignancies in extracutaneous sites has been found, although no separation of BCCs and SCCs was made in these studies [35, 36]. This current finding connects extracutaneous malignancies especially to BCC risk in SCC/PM subjects, as no similar connection was found for SCC/PM risk in subjects with BCC. This association suggests the existence of mutual mechanisms or genetic predisposition behind the carcinogenetic events in the skin and extracutaneous sites. Regarding UV exposure, outdoor or mixed occupation and multiplicity of AKs, both indicating cumulative sun exposure, were independently associated with lower BCC risk, which may be related to life-long, cumulative sun exposure being primarily a risk factor for lesions of the SCC line, rather than BCC [32]. Interestingly, PLR showed no significant association with BCC risk in SCC/PM subjects, unlike in the preceding regression analyses. A possible explanation could be the similarity of the two groups with regard to actinic damage, as both groups had SCC and/or PM as well as similar PAASI scores and sunburn history and may consequently exhibit similar levels of UV-induced skin inflammation.

In conclusion, the most evident finding of this study was the wide spectrum of PM and malignant lesions within subjects. In addition, the study suggests that there may be subjects with a certain type of skin malignancy, such as a group with BCC, SCC/PM or MM only, although the difference in age emphasizes the need for further follow-up. The PLR, previously used in the estimation of systemic disease-related inflammation and cancer prognosis, showed significant associations with the increased risk of further skin lesions of KC or SCC/PM in subjects with MM or BCC, respectively. However, future research is needed to solidify the significance of this marker in clinical skin cancer risk estimation. Another interesting finding was that AMS was associated with a reduced risk of SCC/PM in subjects with BCC. The basis of this interaction remains unknown, but the explanation could lie in genetic susceptibility or other common factors. Nevertheless, the findings highlight the importance of patient education on the risk of further skin malignancies after the first skin cancer.

## Data Availability

The datasets analyzed during the current study are not publicly available due to the risk of compromising patient safety and privacy but are available from the corresponding author on reasonable request.
